# Supplementary material for: DRB1, DRB2 and DRB4 Are Required for an Appropriate miRNA-Mediated Molecular Response to Osmotic Stress in Arabidopsis thaliana
Source: Int J Mol Sci. 2024 Nov 22;25(23):12562. doi: 10.3390/ijms252312562 (PMC11641234; doi:10.3390/ijms252312562)
Supplement: Supplementary file 1 [file ijms-25-12562-s001.zip › ijms-3295658-supplementary.pdf]

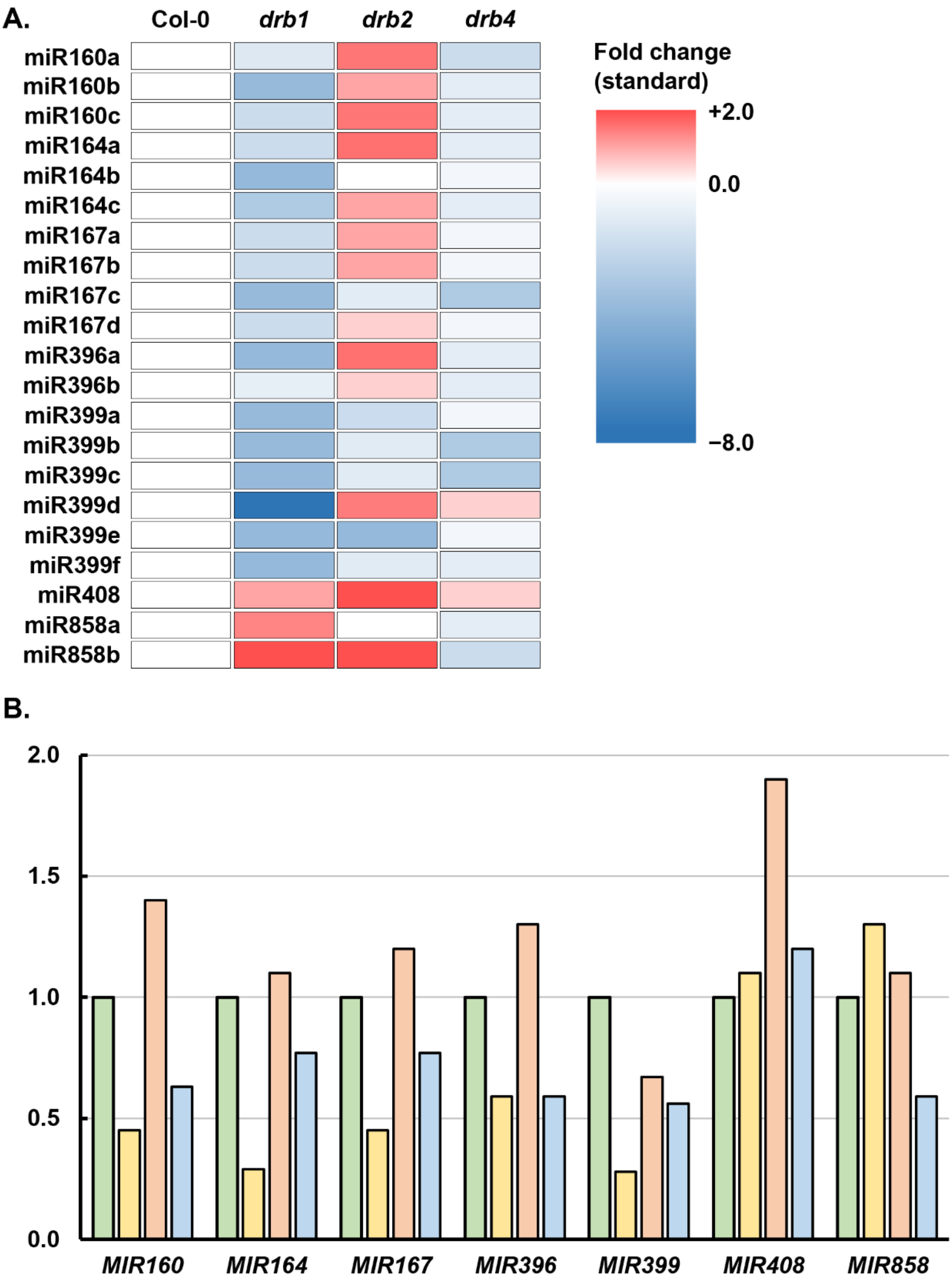

Figure S1. Profiling of the miRNA abundance trends in 15-day-old control grown Col-0, *drb1*, *drb2* and *drb4* seedlings. (**A**) The abundance trend of each individual member of the *MIR160*, *MIR164*, *MIR167*, *MIR396*, *MIR399*, *MIR408* and *MIR858* gene families in 15-day-old control grown Col-0, *drb1*, *drb2* and *drb4* whole seedlings. The shading intensity of each tile of each column in the heat map represents the degree of elevated (red colored shading) or reduced (blue colored shading) abundance (presented as a standard fold change) of each member of the seven *MIR* gene families selected for further analysis in the *drb1*/Ns, *drb2*/Ns and *drb4*/Ns samples compared to the Col-0/Ns sample. (**B**) Standard fold change assessment of the abundance of the 'summed total' of the members of the seven *MIR* gene families selected for further analysis in 15-day-old control grown *drb1* (yellow columns), *drb2* (orange columns) and *drb4* (blue columns) seedlings compared to the sequence data obtained for the Col-0/Ns sample (green columns).

Table S1. Determination of *MIR* gene family member abundance in 15-day-old control grown and mannitol-stressed Col-0, *drb1*, *drb2* and *drb4* seedlings. The abundance of each family member was summed together to allow for the subsequent comparison between the total sRNA-Seq read numbers for the *MIR160*, *MIR164*, *MIR167*, *MIR396*, *MIR399*, *MIR408* and *MIR858* gene families in the Col-0/Ns, *drb1*/Ns, *drb2*/Ns and *drb4*/Ns samples and the Col-0/Mann, *drb1*/Mann, *drb2*/Mann and *drb4*/Mann samples. In the Table a (+) symbol denotes elevated miRNA abundance (as a fold change) in the mannitol-stressed sample compared to the control sample, and a (-) symbol denotes reduced miRNA accumulation (as a fold change) in the mannitol-stressed sample compared to the control sample.

MIR160	***MIR* gene family member sRNA-Seq reads**						**Total Family Reads**	**Fold change (+/-)**
	**miR160a**	**miR160b**	**miR160c**					
Col-0/Ns	204	234	401				*839*	**+3.2**
Col-0/Mann	710	646	1339				*2695*	
drb1/Ns	129	64	194				*387*	**+1.5**
drb1/Mann	198	85	289				*572*	
drb2/Ns	285	301	579				*1165*	**+1.2**
drb2/Mann	326	343	724				*1393*	
drb4/Ns	100	135	295				*530*	**+2.5**
drb4/Mann	281	355	699				*1335*	
MIR164	***MIR* gene family member sRNA-Seq reads**						**Total Family Reads**	**Fold change (+/-)**
	**miR164a**	**miR164b**	**miR164c**					
Col-0/Ns	94	677	175				*946*	**+1.8**
Col-0/Mann	364	752	578				*1694*	
drb1/Ns	48	164	65				*277*	**+1.7**
drb1/Mann	153	203	111				*467*	
drb2/Ns	159	654	219				*1032*	**+1.2**
drb2/Mann	283	590	385				*1258*	
drb4/Ns	67	570	106				*743*	**+1.7**
drb4/Mann	260	643	376				*1279*	
MIR167	***MIR* gene family member sRNA-Seq reads**						**Total Family Reads**	**Fold change (+/-)**
	**miR167a**	**miR167b**	**miR167c**	**miR167d**				
Col-0/Ns	3229	2896	1326	1890			*9341*	**+2.9**
Col-0/Mann	9467	8521	3071	5913			*26972*	
drb1/Ns	1552	1510	366	849			*4277*	**+1.3**
drb1/Mann	2047	1852	221	1297			*5417*	
drb2/Ns	4108	3791	1076	2136			*11111*	**+1.4**
drb2/Mann	5540	4965	1478	3332			*15315*	
drb4/Ns	2597	2299	554	1486			*6936*	**+2.2**
drb4/Mann	5337	4641	1542	3444			*14964*	
MIR396	***MIR* gene family member sRNA-Seq reads**						**Total Family Reads**	**Fold change (+/-)**
	**miR396a**	**miR396b**						
Col-0/Ns	4316	7784					*12100*	**+2.8**
Col-0/Mann	12785	21346					*34131*	
drb1/Ns	1078	5840					*6918*	**-1.1**

drb1/Mann	897	5372					*6269*	
drb2/Ns	6480	9630					*16110*	**+1.3**
drb2/Mann	7623	12862					*20485*	
drb4/Ns	2750	4293					*7043*	**+2.3**
drb4/Mann	6324	10211					*16535*	
MIR399	***MIR* gene family member sRNA-Seq reads**						**Total Family Reads**	**Fold change (+/-)**
	**miR399a**	**miR399b**	**miR399c**	**miR399d**	**miR399e**	**miR399f**		
Col-0/Ns	386	322	341	8	4	25	*1086*	**+3.2**
Col-0/Mann	1306	999	1020	33	11	56	*3425*	
drb1/Ns	104	96	98	1	0	7	*306*	**-2.1**
drb1/Mann	66	40	34	0	0	4	*144*	
drb2/Ns	210	237	240	12	1	18	*718*	**+1.4**
drb2/Mann	326	215	231	18	4	30	*824*	
drb4/Ns	317	120	126	9	3	18	*593*	**+2.2**
drb4/Mann	605	311	305	20	3	44	*1288*	
MIR408	***MIR* gene family member sRNA-Seq reads**						**Total Family Reads**	**Fold change (+/-)**
	**miR408**							
Col-0/Ns	2242						*2242*	**+2.2**
Col-0/Mann	4828						*4828*	
drb1/Ns	2436						*2436*	**+1.8**
drb1/Mann	4377						*4377*	
drb2/Ns	4322						*4322*	**-2.4**
drb2/Mann	1791						*1791*	
drb4/Ns	2657						*2657*	**-1.1**
drb4/Mann	2475						*2475*	
MIR858	***MIR* gene family member sRNA-Seq reads**						**Total Family Reads**	**Fold change (+/-)**
	**miR858a**	**miR858b**						
Col-0/Ns	79	4					*83*	**+4.0**
Col-0/Mann	301	27					*328*	
drb1/Ns	101	7					*108*	**-3.0**
drb1/Mann	34	2					*36*	
drb2/Ns	82	7					*89*	**+1.9**
drb2/Mann	162	10					*172*	
drb4/Ns	46	3					*49*	**+4.0**
drb4/Mann	181	15					*196*	

Table S2. Sequences of the DNA oligonucleotides used in this study.

Target analyzed	Oligonucleotide name	Oligonucleotide sequence (5′ to 3′)
miRNA-specific cDNA synthesis and RT-qPCR analysis		
miR160	p-mir160-RTF	CGCCTGACAGAAGAGAGTGAGCAC
	p-mir160-RTR	GTCGTATCCAGTGCAGGGTCCGAGGTATTCGCACTGGATACGACGTGCTC
miR164	p-mir164-RTF	GGCTGGAGAAGCAGGGCACGTGCA
	p-mir164-RTR	GTCGTATCCAGTGCAGGGTCCGAGGTATTCGCACTGGATACGACTGCACG
miR167	p-mir167-RTF	CGCTGAAGCTGCCAGCATGATCTA
	p-mir167-RTR	GTCGTATCCAGTGCAGGGTCCGAGGTATTCGCACTGGATACGACCGGCAA
miR396	p-mir396-RTF	GCGCGTTCCACAGCTTTCTTGAAC
	p-mir396-RTR	GTCGTATCCAGTGCAGGGTCCGAGGTATTCGCACTGGATACGACAAGTTC
miR399	p-mir399-RTF	GCATGCCAAAGGAGATTTGCCCTG
	p-mir399-RTR	GTCGTATCCAGTGCAGGGTCCGAGGTATTCGCACTGGATACGACCAGGGC
miR408	p-mir408-RTF	ACGACAGGGAACAAGCAGAGCATG
	p-mir408-RTR	GTCGTATCCAGTGCAGGGTCCGAGGTATTCGCACTGGATACGACCATGCT
miR858	p-mir858-RTF	GGCGTTTCGTTGTCTGTTCGACCTT
	p-mir858-RTR	GTCGTATCCAGTGCAGGGTCCGAGGTATTCGCACTGGATACGACAAGGTC
Generic Stem-loop	p-generic-STL	CCAGTGCAGGGTCCGAGGTA
snoR101	p-snor101-RTF	CTTCACAGGTAAGTTCGCTTG
	p-snor101-RTR	AGCATCAGCAGACCAGTAGTT
High molecular weight cDNA synthesis and RT-qPCR analysis		
Ath-DCL1	p-dcl1-RTF	AATGGGCATCAGCCGTTTACGAGA
	p-dcl1-RTR	AAATCTCTTTGCATGAGCCGGTCC
Ath-DRB1	p-drb1-RTF	ATGACCTCCACTGATGTTTCC
	p-drb1-RTR	TGCTAATTCCCGGAGAGC
Ath-DRB2	p-drb2-RTF	ATGTATAAGAACCAGCTACAAGAGTTG
	p-drb2-RTR	CAGCAGCAGAGTGTTCAGC
Ath-DRB4	p-drb4-RTF	AAATGGGAACTCGAACCAGA
	p-drb4-RTF	CCACCTTGGAAGAAGGTTGA
Ath-P5CS1	p-p5cs1-RTF	GTTTTTGAATCCCGACCTGA
	p-p5cs1-RTR	TTACCCCCAACAGTCTCTGG
Ath-UBI10	p-ubi10-RTF	GGCCTTGTATAATCCCTGATGAATAAG
	p-ubi10-RTR	AAAGAGATAACAGGAACGGAAACATA
Ath-ARF8	p-arf8-RTF	GGTTGGGCGTTCATTAGACA
	p-arf8-RTR	ATGTACCAAACGTTATTCACA
Ath-ARF17	p-arf17-RTF	CGAGTCAAGATGGCTATGGA
	p-arf17-RTR	CATCCCATGTGATCTGAAGC
Ath-CUC1	p-cuc1-RTF	CTTCTTCTTCTGCCGTCACC
	p-cuc1-RTR	GAGCGGGAAGGAATGTATGA
Ath-GRF7	p-grf7-RTF	CATCCCCCACCGTTAGATCG
	p-grf7-RTR	TGCTTCCATGCTTCCGACAT
Ath-PHO2	p-pho2-RTF	ACCGTTTCTCATCAAGGCGT
	p-pho2-RTR	GTGCCCGTCCACCATAAGAA
Ath-LAC3	p-lac3-RTF	CCGTTCGACAACACAACCAC
	p-lac3-RTR	GACTGGGAAAACAGGAGCGA
Ath-ERF7	p-erf7-RTF	CCGTGGCATAGAAGCAAAGT
	p-erf7-RTR	CGGTGAGTGGTTTTGTTGTG
poly-A tail	p-oligo-dT18	TTTTTTTTTTTTTTTTTT
